# Waist-Bands for Fat Men

**Published:** 1921-04-30

**Authors:** 


*To the Editor of* The Hospital.

### Waist-Bands for Fat Men.

*To the Editor of* The Hospital.

Sir,—The experience of the manual worker who discovered the virtues of a Sam Browne belt, and of whose fitness you recounted in your last issue, is surely not unusual. What is at least controversial is the suggestion that he became fit because, belt-fastened, he could—or rather had to—breathe with his chest.

Where the belt comes in—and on this I think, among other authorities, I might quote Sir Arbuthnot Lane—is prevention of many things, among others a visceroptosis or downward sagging of the abdominal contents, a tendency for too great a volume of blood to accumulate in this particular area, and a general inefficiency of the gastro-intestinal system owing to its displacement and loss of tone.

I hardly think that the surgical-appliance maker would credit your manual worker with the making of a discovery, but no doubt he himself greatly appreciated any permanent benefit from his war service, indirect though it happened to be. All the same, he is an optimist and perhaps a little unobservant, if he believes that a Sam Browne is a permanent preventive against adiposity.—Yours, etc.,

Falex.

